# High expression of nectin-1 indicates a poor prognosis and promotes metastasis in hepatocellular carcinoma

**DOI:** 10.3389/fonc.2022.953529

**Published:** 2022-08-18

**Authors:** Xuequan Wang, Ziming Xing, Huazhong Chen, Haihua Yang, Qiupeng Wang, Tongjing Xing

**Affiliations:** ^1^ Taizhou Hospital of Zhejiang Province Affiliated to Wenzhou Medical University, Linhai, China; ^2^ Academy of Medical Engineering and Translational Medicine, Tianjin University, Tianjin, China

**Keywords:** hepatocellular carcinoma, nectin-1, prognostic, cell proliferation, metastasis

## Abstract

**Objectives:**

Nectins are a new class of cell-adhesion molecules that play an important role in tumorigenesis and disease progression. The aim of this study was to investigate the prognostic and pathogenetic roles of nectins in hepatocellular carcinoma (HCC).

**Methods:**

The expression levels of the nectin family in HCC and their role in prognosis were analyzed by bioinformatics analysis based on The Cancer Genome Atlas (TCGA) liver hepatocellular carcinoma database. The correlations between nectins and immune cells were analyzed using TIMER. The functional enrichment of the nectin-1 coexpression network was evaluated in TCGA cohort, and the expression levels of nectin-1 were detected by immunohistochemistry and Western blot analysis. A Transwell kit was used for cell migration experiments. Cell proliferation was analyzed using Cell Counting Kit-8.

**Results:**

The expression levels of nectin-1 protein in the cancer tissues of 28 patients with HCC were higher than those in paracancerous tissues. The Kaplan–Meier plotter analysis showed that the high expression of all nectin family numbers was related to the poor prognosis of HCC patients. The abnormal expression of nectin-1 effectively distinguished the prognosis at different stages and grades of HCC. The high expression of 17 methylation sites of the nectin-1 gene was related to the high overall survival of HCC patients. Kyoto Encyclopedia of Genes and Genomes analysis of genes negatively correlated with nectin-1, revealing their close relation to the regulation of the immune-effector process. Pearson’s correlation analysis showed that nectin-1 was significantly positively correlated with multiple immune genes and B cells, CD4^+^ T cells, macrophages, neutrophils, and dendritic cell infiltration. Cell proliferation of the knockdown (KD) group decreased significantly compared to the NC-KD group. The number of metastatic cells in the KD group decreased significantly compared to that in the NC-KD group.

**Conclusions:**

Abnormal expression of nectins and multiple methylation sites closely correlates with poor prognosis in HCC patients. Nectins are related to immune cell infiltration and immune-related genes. In particular, nectin-1 can promote the proliferation and migration of liver cancer cells and distinguish the prognosis at different stages and grades of HCC. Nectin-1 might be a new potential molecular marker for prognostic evaluation and also a therapeutic target for HCC.

## Introduction

Hepatocellular carcinoma (HCC) is the second leading cause of cancer mortality and the sixth most common cancer worldwide (1). Surgical resection is the main treatment strategy for early-stage HCC. However, the recurrence rate 5 years after an operation is >70%. Most cases of HCC are diagnosed at an intermediate or advanced stage. Although great progress has been made in molecular and immune-targeted therapy in recent years, the 5-year survival rate of HCC is still relatively low (2). The prognosis of HCC patients depends not only on tumor characteristics but also on etiology, liver function, and individual differences. Some prognostic models and markers of HCC have been proposed and applied in clinical settings (3). However, it is still necessary to find new molecular markers to predict the prognosis or therapeutic effect of HCC because it is a highly heterogeneous tumor.

The nectin family, also known as the polyvirus receptor-related protein (pvrl) family, is a new class of cell-adhesion molecules belonging to the immunoglobulin gene superfamily, which takes part in immune response and immunoregulation. Nectin has four isoforms: nectin-1 (pvrl1), nectin-2 (pvrl2), nectin-3 (pvrl3), and nectin-4 (pvrl4). Nectins are calcium-independent intercellular-adhesion molecules that can interact with cadherins to form intercellular connections (4). Human nectin-1, nectin-2, and nectin-3 are expressed by fibroblasts, epithelial cells, and neuronal cells, while human nectin-4 is mainly expressed during embryonic development and in tumor tissues (5). Nectins not only mediate adhesion between cells but also have a great impact on the biological behavior of cells. Abnormal expression of nectins can lead cells to lose their original stability, enhance cell proliferation activity and migration ability, and promote the occurrence and development of malignant tumors (6).

Recently, the role of nectins in tumorigenesis and tumor progression has attracted considerable research attention. Some studies have found that the abnormal expression of nectin-4 is closely related to the recurrence, metastasis, and overall survival (OS) rate of many tumors, including HCC (7–13). However, there have been few reports on the roles of nectin-1, nectin-2, and nectin-3 in cancer development and prognosis (14–16). In particular, their roles in HCC have not been reported. In this study, the expression levels of the nectin gene family in HCC and their role in prognosis were analyzed by bioinformatics analysis. In particular, the effects of nectin-1 on the biological characteristics of liver cancer cells were investigated. The expression levels of nectin-1 protein in HCC were detected by immunohistochemistry (IHC).

## Methods

### Data processing and analysis of differences in nectin expression in HCC

Gene-expression data and DNA-methylation data were downloaded from the University of California, Santa Cruz, Xena GDC Cancer Genome Atlas (TCGA) liver hepatocellular carcinoma (LIHC) datasets (https://xenabrowser.net/datapages/). Differences in the expression levels of nectins between 424 HCC tissues and 50 adjacent nontumor tissues were analyzed by the Wilcoxon test in paired and unpaired groups based on HiSeq (Illumina, San Diego, CA, USA) RNA-sequencing data in fragments per kilobase per million mapped reads (FPKM) form. The expression patterns of nectins were identified on the IHC images of LIHC and normal specimens from the Human Protein Atlas (http://www.proteinatlas.org/) database.

### Sample collection

Paraffin-embedded specimens of cancer tissues and paired adjacent tissues of 28 HCC patients were collected from January 2019 to November 2021 in Taizhou Hospital Affiliated with Wenzhou Medical University. All pathological sections were diagnosed by two senior pathologists. Diagnosis and treatment of HCC were performed in accordance with the guidelines for the diagnosis and treatment of primary liver cancer (2019 version) (Bureau of Medical Administration, National Health Commission of the People’s Republic of China) (17). Hepatitis B virus-related HCC patients with an initial diagnosis who underwent surgical resection were included. The baseline data of patients are shown in **Table 1**. We excluded patients with HCC caused by chronic hepatitis C, alcoholic liver disease, autoimmune liver disease, or drug-induced liver disease; patients with HCC who received radiotherapy, chemotherapy, or interventional therapy before the operation; and patients with HCC combined with cholangiocarcinoma and other organ tumors. The experimental protocol was approved by the Ethical Commission of Taizhou Hospital. The samples collected for research purposes were clearly stated in the signed informed consent form that they could be used for subsequent clinical research. All data were fully anonymized before we accessed them.

**Table 1:** Baselines of patients with hepatocellular carcinoma.

HCC	
**Sex (m/f)**	24/4
**Age (year)**	59 (40–78)
**ALT (U/L)**	40.2 ± 39.7
**HBV DNA (Log^10^IU/ml)**	3.46 ± 2.28
**TBil (mmol/L)**	18.9 ± 16.9
**ALB (g/L)**	41.5 ± 4.3
**AFP (ng/ml)**	3,517.9 ± 6,254.7

### Survival analysis according to nectin expression in HCC

After grouping TCGA-LIHC patients into a high-expression group and a low-expression group based on the expression level of each nectin, differences in the OS between the low- and high-expression groups were evaluated with the Kaplan–Meier method and the log-rank test while considering the survival information revealed by the “Surv_cutpoint” function in the survminer R package (18). Univariate Cox regression analysis was performed using the “survival” package (19) to investigate the correlation between the OS characteristics of LIHC patients and the expression levels of each nectin. Multivariate Cox regression analysis performed using the “survival” package was carried out to investigate whether these nectins are independent predictors of OS (20).

### Relationship between nectins and clinical staging characteristics of LIHC patients

To explore the relationship between the expression levels of nectins and the clinicopathological characteristics of LIHC patients, information on the age, stage, and histological type of each LIHC patient was also retrieved. All of the LIHC patients were then grouped based on different clinical staging information (T/N/M, grade, stage). Differences in the expression levels of nectins between different clinicopathological stages were analyzed using the Kruskal–Wallis test and presented by the “RainCloudPlots” package (21). Differences in OS between the nectin-1 high- and low-expression groups in different clinicopathological stages were evaluated by Kaplan–Meier survival analysis (22).

### Gene functional enrichment analysis of nectin-1 coexpressed genes

The coexpressed genes of nectin-1 in LIHC were screened by Pearson’s correlation analysis with coefficient cutoff |cor| > 0.3 and *p* < 0.05. The Pearson’s coefficients, combined with *p-*values, were plotted in a volcano plot using the “ggplot2” package (23), and the top 50 negative or top 50 positive nectin-1 coexpressed genes were plotted by the “pheatmap” R package (24). The functional enrichment of nection-1 coexpressed genes was analyzed using Metascape (https://metascape.org/gp/index.html) (22).

### Methylation analysis of nectins in LIHC samples

MEXPRESS (https://mexpress.be/index.html) was used to visualize the expression, methylation, and clinical data of nectins in LIHC (25). DNA-methylation profiles from TCGA for nectins, including 377 patients’ samples in level 3, were extracted based on the Illumina HumanMethylation450 Beadchip and the hg19 GPL16304 legacy annotation file. For each methylation site in nectin-1, the methylation β-value was divided into low and high groups using the survminer package. The significant OS-associated sites for the high- and low-methylation groups were screened by univariate Cox regression analysis. The correlations between the β-values of each nectin-1 methylation site and the FPKM expression value of nectin-1 were calculated using the “corrplot” package (26).

### Analysis of infiltrating immune cells in LIHC tumor microenvironment

Based on Tumor Immune Estimation Resource 2.0 (TIMER 2.0, http://timer.cistrome.org/), which is a novel statistical web resource for the systematic evaluation of the clinical impact of immune cell infiltration in tumors, the levels of six types of immune cells (B cells, CD4^+^ T cells, CD8^+^ T cells, neutrophils, macrophages, and dendritic cells (DCs)) were estimated for each LIHC patient (27). The correlations between the expression levels of nectins and the infiltrating immune cells’ enrichment were calculated using the “corrplot” package (26). The “ggstatsplot” package was then used to create detailed correlation graphics with Pearson’s statistical tests.

### Identification of the relationship between nectin-1 and tumor-infiltrating immune cell–related genes

The tumor-infiltrating immune cell (TIIC)–related genes were collected from a published study that contained 568 immune-related genes for 28 types of specifically labeled TIICs (28). The correlation analysis of nectin-1 and immune-related genes was performed by Pearson’s correlation analysis. Detailed correlations of nectin-1 with the top three negative, top three positive, and three immune checkpoint–related genes were depicted by the “ggstatsplot” package (29).

### Cell culture and infection

Five human hepatoma cell lines, including SK-Hep-1, Hep 3b, RBE, Huh-7, and PLC/PRF/5, were purchased from the Cell Institute of the Shanghai Chinese Academy of Sciences. All of the cells were incubated in DMEM or RPMI 1640 supplemented with 10% fetal bovine serum (FBS) (Gibco Laboratories, Gaithersburg, MD, USA) and were cultured in an incubator at 37°C with 5% CO_2_. The cells were infected with the recombinant lentiviral vector labeled with green fluorescence to establish nectin-1–knockdown (KD) and nectin-1–overexpression (OE) cells, in accordance with the manufacturer’s instructions (GeneChem Corporation, Shanghai, China). Briefly, the cells were digested at the logarithmic growth phase, resuspended at a concentration of 4 × 10^4^ cells/ml, and seeded in six-well plates. At 20% confluence, the cells were infected with the recombinant lentivirus for 16 h. Small interfering RNAs and an OE-specific lentiviral vector for nectin-1 were purchased from GenePharma (Shanghai, China), and the detailed KD sequences were as follows: nectin-1 RNAi (KD-1) sense: AGAACAGAACCCTCTTCTT; nectin-1 RNAi (KD-2) sense: CACTCTCAACGTGCAGTAT; nectin-1 RNAi (KD-3) sense: AGTGTGGTATCCTGGGAAA; and NC-RNAi sense: TCTGTATAAT CGGCTGGTT.

### Detection of nectin-1 expression by fluorescence quantitative polymerase chain reaction

Total RNA was extracted from cells using TRIzol reagent (Invitrogen, Carlsbad, CA, USA). Complementary DNA was obtained by reverse transcription using the Promega M-MLV kit (Promega Corp. Madison, WI, USA). The polymerase chain reaction (PCR) reaction was carried out on the real-time fluorescent quantitative PCR detector (LightCycler 480 II, Roche Diagnostic Corp., Basel, Switzerland). The reaction conditions of fluorescence quantitative PCR (Promega Corp., Madison, WI, USA) were 95°C for 30 s, then 40 cycles of 95°C for 5 s and 60°C for 30 s. The nectin-1 primers were designed and synthesized by Shanghai Genechem Co., Ltd. The sequences of the primers were as follows: forward, 5′-TACATCTGCGAG TTTGCTAC-3′; reverse, 5′-CTCACCTTTTAACCGAGTTT-3′. β-Actin (ACTB) was used as an internal reference: forward, 5′-GCGTGACATTAAGGAGAAGC-3′; reverse, 5′-CCACGTCACACTTCAT GATGG-3′.

### Cell proliferation assay

SK-Hep-1 cells were infected with lentivirus containing nectin-1 interference or RNA OE. After 5 days of culture, the cells in the logarithmic growth phase were digested with trypsin (Gibco Laboratories), resuspended in a complete medium (Corning, Inc., Corning, NY, USA), and counted. Then, after seeding 100 μl/well (1.5 × 10^3^ cells) in 96-well plates, 10 μl of Cell Counting Kit (CCK)-8 reagent (Dojindo Laboratories, Kumamoto, Japan) was added to each well at 4 h before the end of culture. The optical density (OD) value of each well was detected at 450 nm (Infinite M2009PR; Tecan, Männedorf, Switzerland).

### Cell migration assay

A transwell kit (Corning Costar, Cambridge, MA, USA) was used for the cell migration experiment. The chamber was placed on a 24-well plate. We seeded 100 µl of SK-Hep-1 cell suspension (0% FBS DMEM) into the upper chamber (1 × 10^4^/well) and 600 µl of 30% FBS medium—into the bottom chamber. The nonmetastatic cells in the chamber were removed after culturing in a 37°C incubator for 16 h. The chamber was fixed in 4% paraformaldehyde for 30 min. The transferred cells were stained with Giemsa staining solution (Sigma-Aldrich, St. Louis, MO, USA). Each Transwell cell was photographed with a microscope (IX71; Olympus Corporation, Tokyo, Japan). We randomly selected visual fields and counted cells at ×200 magnification. The number of transferred cells per field in each group was calculated.

### Western blot analysis

The SK-Hep-1 cells were digested with 0.25% trypsin ethylenediaminetetraacetic acid, centrifuged, and lysed with cold radioimmunoprecipitation assay lysis buffer (Beyotime Biotechnology, Shanghai, China). The supernatant was collected and stored at −80°C. Next, the sample (8 µl) and 5× sodium dodecyl sulfate-polyacrylamide gel electrophoresis (SDS-PAGE) loading buffer (2 µl) were added, heated at 100°C for 5 min, and centrifuged. The samples were loaded onto 8% SDS-PAGE for electrophoresis. The polyvinylidene difluoride membrane was soaked for 10 min and transferred by the wet transfer method. Rabbit anti-nectin-1 polyclonal antibody (Proteintech Corporation, Rosemont, IL, USA) was added and incubated overnight, followed by incubation with horseradish peroxidase-conjugated secondary antibody for 1.5 h. Chemical luminescence detection was performed by the FR-1800 fluorescence biological image analysis system (Shanghai Furi Science & Technology Co., Ltd., Shanghai, China). Gel-Pro analyzer software was used for analysis and processing.

### Immunohistochemistry

Immunohistochemistry was performed using the conventional streptavidin-peroxidase method. The paraffin-embedded tissue sections were dewaxed to hydration and antigen-repaired with high pressure. Endogenous peroxidase was blocked with 3% hydrogen peroxide at room temperature for 15 min, and the sections were incubated with 5% bovine serum albumin (BSA) for 30 min. Next, the sections were incubated with nectin-1 antibody (Proteintech Corporation) at 4°C overnight. The secondary antibody was added and incubated for 2.5 h. A streptomyces antibiotic peroxidase solution was added and incubated at room temperature for 20 min. The tissue sections were counterstained with hematoxylin and differentiated with hydrochloric acid ethanol. Positive nectin-1 staining was observed under DM 500 microscope (Leica, Wetzlar, Germany).

## Results

### Messenger RNA- and protein-expression levels of the nectin family in HCC patients

**Figure 1:**
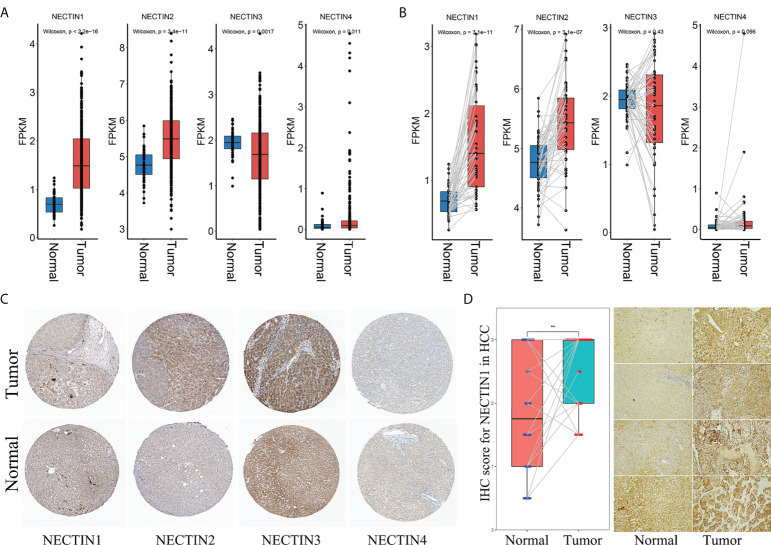
Different expressions of nectin-1, nectin-2, nectin-3, and nectin-4 genes and proteins in HCC tissue. **(A)** Differential expression of nectin-1, nectin-2, nectin-3, and nectin-4 genes in TCGA-LIHC patients. **(B)** Differential expression of nectin-1, nectin-2, nectin-3, and nectin-4 genes in TCGA-LIHC HCC paired tissues. **(C)** Typical pictures of nectin-1, nectin-2, nectin-3, and nectin-4 protein expression from the HPA database. **(D)** The expression of the nectin-1 protein in cancer tissues and paracancerous tissues of 28 patients with HCC. **: P<0.05.

Levels of nectin family messenger RNAs (mRNAs) were obtained from TCGA-LIHC database. The results showed that the expression levels of nectin-1 and nectin-2 genes in HCC tissues were higher compared to those in normal tissues adjacent to cancer from all patients (*p* < 0.001). Also, the expression level of the nectin-3 gene was lower and that of nectin-4 was higher in HCC tissues compared to in normal tissues adjacent to cancer from all patients (*p* < 0.05) (**Figure 1A**). Meanwhile, the expression levels of nectin-1 and nectin-2 genes were higher in HCC tissues compared to in normal tissues adjacent to cancer from paired HCC patients (*p* < 0.001), while no significant differences were found in the expression levels of nectin-3 and nectin-4 between the paired HCC tissues (**Figure 1B**). Protein-expression levels of the nectin family in HCC and normal liver tissues were analyzed using data from the Human Protein Atlas (HPA) database. The results showed that the expression levels of nectin-1 and nectin-4 proteins were low in HCC and normal liver tissues; meanwhile, the expression levels of nectin-2 protein were medium in both HCC and normal liver tissues, and the expression levels of nectin-3 protein were high in HCC but low in normal liver tissues, respectively (**Table 2**). The representative images of the nectin family proteins in HCC and normal tissues are shown in **Figure 1C**. The expression levels of nectin-1 protein in cancer tissues of 28 patients with HCC (2.55 ± 0.61) were higher than those in paracancerous tissues (1.82 ± 0.94) according to IHC (*t* = 3.44, *p* < 0.001) (**Figure 1D**).

**Table 2:** Immunohistochemical stains of nectin protein obtained from the Human Protein Atlas in normal liver tissues and hepatocellular carcinoma tissues.

Genes	Normal	Cancer (cases)				Antibody
		High	Medium	Low	Not detected	
Nectin 1	Low	0	0	2	5	HPA007730
Nectin 2	Medium	1	5	0	0	HPA012759
Nectin 3	Medium	1	5	0	0	HPA011038
Nectin 4	Low	0	0	4	1	HPA010775

### Correlations between the expression levels of nectin family genes and the prognosis of HCC

**Figure 2:**
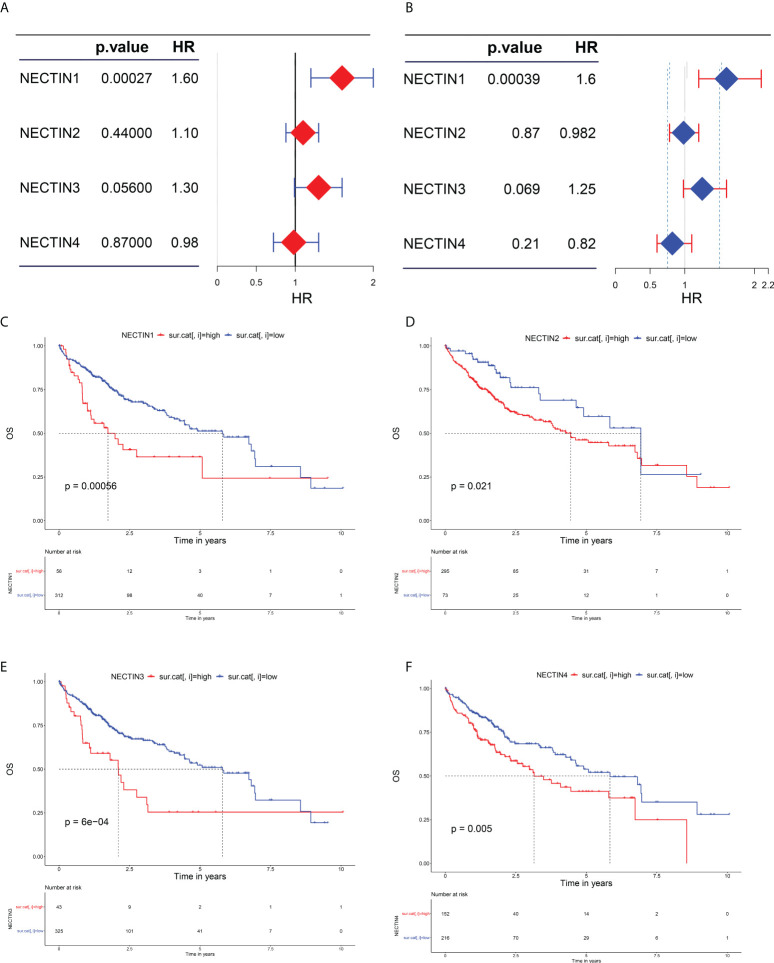
Evaluation of nectin-1, nectin-2, nectin-3, and nectin-4 genes and prognosis in patients with HCC. **(A)** Univariate analysis of nectin-1, nectin-2, nectin-3, and nectin-4 and overall survival rate of HCC patients plotted by “forestplot.” **(B)** Multivariate analysis of nectin-1, nectin-2, nectin-3, and nectin-4 and overall survival rate of HCC patients plotted by “forestplot.” **(C)** K-M plotter of nectin-1 gene expression and OS in HCC patients. **(D)** K-M plotter of nectin-2 gene expression and OS in HCC patients. **(E)** K-M plotter of nectin-3 gene expression and OS in HCC patients. **(F)** K-M plotter of nexin-4 gene expression and prognosis of HCC patients.

The effects of abnormal expression of nectin family genes on the OS rate of HCC patients were evaluated by the univariate regression analysis. The results showed that the high expression level of nectin-1 was related to a high OS rate in HCC patients (hazard ratio (HR), 1.6; 95% confidence interval (CI), 1.2–2; *p* = 0.00027). The low expression levels of nectin-3 tended to be related to the high OS rate of HCC patients (HR, 1.3; 95% CI, 0.99–1.6; *p* = 0.056). The expression levels of nectin-2 (HR, 1; 95% CI, 0.88–1.3; *p* = 0.44) and nectin-4 (HR, 0.98; 95% CI, 0.72–1.3; *p* = 0.87) had no significant association with the OS rate of HCC patients (**Figure 2A**). The multivariate analysis showed that the high OS rate of HCC patients was independently associated with the expression of nectin-1 in HCC and was not affected by the expression of other nectin family members (HR, 1.6; 95% CI, 1.2–2.1, *p* = 0.00039) (**Figure 2B**). According to the expression levels of the nectin family genes, patients with HCC were divided into high- and low-expression groups. Kaplan–Meier plotter analysis showed that the high expression levels of all nectin family numbers were related to the poor prognosis of HCC patients (**Figures 2C–F**).

### Differences in expression and prognostic significance of the nectin family in different stages or grades of HCC

**Figure 3:**
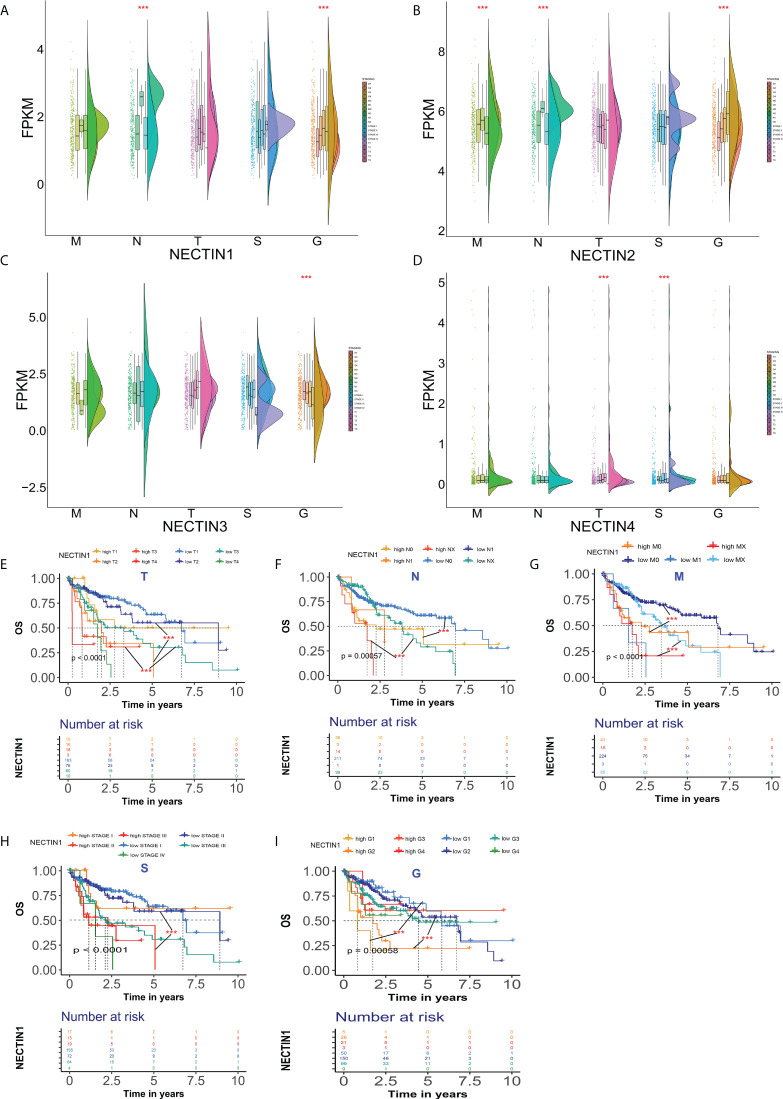
Differential expression and prognosis of nectin-1, nectin-2, nectin-3, and nectin-4 in different stages and TNM stages of HCC. **(A)** Differential expression of nectin-1 in patients with different stages of HCC analyzed by the “rainclouds” package. **(B)** Differential expression of nectin-2 in patients with different stages of HCC analyzed by the “rainclouds” package. **(C)** Differential expression of nectin-3 in patients with different stages of HCC analyzed by the “rainclouds” package. **(D)** Differential expression of nectin-4 in patients with different stages of HCC analyzed by the “rainclouds” package. **(E)** Nectin-1 and prognosis of T-stage of HCC patients. **(F)** Nectin-1 and prognosis of N-stage of HCC patients. **(G)** Nectin-1 and prognosis of M-stage of HCC patients. **(H)** Nectin-1 and prognosis at different stages of HCC patients. **(I)** Nectin-1 and prognosis at different grades of HCC patients. ***P<0.01.

The expression levels of the nectin family members in different stages and TNM stages of HCC were analyzed. The results showed that there were significant differences in the expression levels of nectin-1 and nectin-2 at different grades of HCC compared to normal tissues. There were also significant differences in the N stage, but there was no significant difference in the T stage (**Figures 3A, B** **)**. The expression levels of nectin-3 were significantly different among different grades of HCC, but there were no significant differences among T, N, and M stages (**Figure 3C**). The expression levels of nectin-4 significantly differed between different grades and T stage; no significant differences were found between the N and M stages of HCC (**Figure 3D**). The Kaplan–Meier plotter analysis showed that the abnormal expression of nectin-1 could be used to evaluate the prognosis of HCC patients with different TNM stages (**Figures 3E–G**); in other words, the abnormal expression of nectin-1 effectively distinguished the prognosis at different stages and grades of HCC patients (**Figures 3H, I** **)**.

### Expression of nectin family DNA methylation in HCC tissue samples and its prognostic correlation

**Figure 4:**
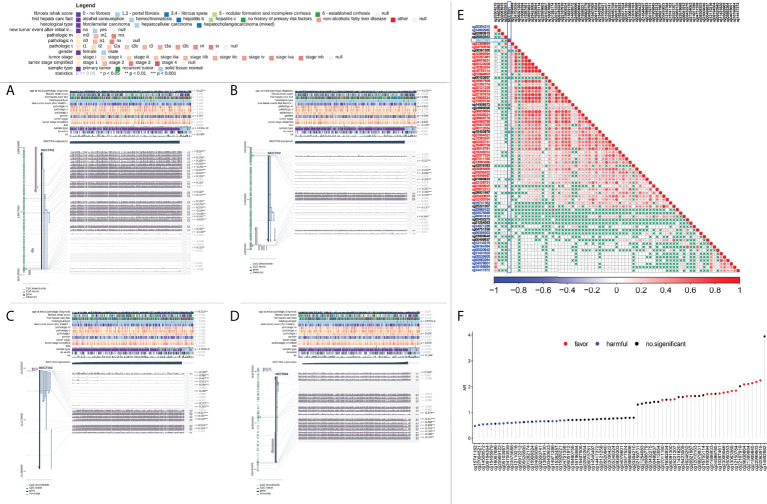
Analysis of DNA methylation of nectin-1, nectin-2, nectin-3, and nectin-4 genes and prognosis of HCC. **(A)** DNA methylation of nectin-1 by MEXPRESS. **(B)** DNA methylation of nectin-2 by MEXPRESS. **(C)** DNA methylation of nectin-3 by MEXPRESS. **(D)** DNA methylation of nectin-4 by MEXPRESS. **(E)** Correlation between nectin-1 gene expression and methylation sites. **(F)** Correlation between methylation sites of nectin-1 gene and OS of patients with HCC.

The correlation analysis between DNA methylation of the nectin family genes in HCC tissues and clinical indicators was carried out using TCGA-LIHC methylation data. The results showed that the methylation of nectin-1 correlated with the OS of HCC patients (*r* = −0.100, *p* < 0.05), while the methylation of nectin-4 significantly correlated with the OS of HCC patients (*r* = −0.104, *p* < 0.05). Meanwhile, the DNA methylation of nectin-2 and nectin-3 genes did not significantly correlate with the OS of HCC patients (**Figures 4A–D**). The relationship between DNA-methylation sites of the nectin gene family and the OS rate of HCC patients was analyzed by Cox regression, and the results showed that nine methylation sites of the nectin-1 gene closely correlated with the OS rate of HCC patients. However, nectin-2, nectin-3, and nectin-4 genes had relatively fewer significant OS-related methylation sites (**Table 3**). The correlation analysis between the nectin-1 DNA-methylation sites and its expression showed 39 positive correlations and 17 negative correlations (**Figure 4E**). The univariate regression analysis showed that the high expression of 17 methylation sites of the nectin-1 gene was related to the high OS of HCC patients. Separately, the high expression of 19 methylation sites was related to the low OS of HCC patients (**Figure 4F**).

**Table 3:** Analysis of nectin-1, nectin-2, nectin-3, and nectin-4 gene methylation sites and overall survival rate of HCC patients.

Gene	Methylation sites	HR	(95% CI for HR)	*p*-value
Nectin1	cg03554194	12.7	(2.54–63.8)	0.00199
Nectin1	cg04677623	0.000171	(7.67*e*−08–0.38)	0.0274
Nectin1	cg05103957	1.07*E*−09	(1.62*e*−18–0.706)	0.0462
Nectin1	cg07704136	9.65*E*−18	(1.52*e*−28–6.13*e*−07)	0.00202
Nectin1	cg08889687	0.357	(0.162–0.786)	0.0106
Nectin1	cg10178308	0.00424	(0.000111–0.162)	0.00329
Nectin1	cg15741162	0.101	(0.022–0.46)	0.00306
Nectin1	cg18709144	0.0116	(0.000701–0.191)	0.00182
Nectin1	cg26921987	13800	(2.04–93800000)	0.0341
Nectin2	cg19522294	5.52*E*−11	(5.08*e*−18–0.000599)	0.00427
Nectin3	cg02882264	34	(2.83–409)	0.00544
Nectin3	cg23009046	18.3	(1.73–194)	0.0157
Nectin4	cg02018544	0.049	(0.00407–0.591)	0.0176
Nectin4	cg04373596	3.5*E*−34	(4.63*e*−62–2.65*e*−06)	0.0187
Nectin4	cg17833106	0.14	(0.0209–0.939)	0.0429

Raw data source: file://TZYY-20210817WN/raw%20data.

### Correlation and Kyoto Encyclopedia of Genes and Genomes analysis between nectin-1 and coexpressed genes in HCC tissue samples

**Figure 5:**
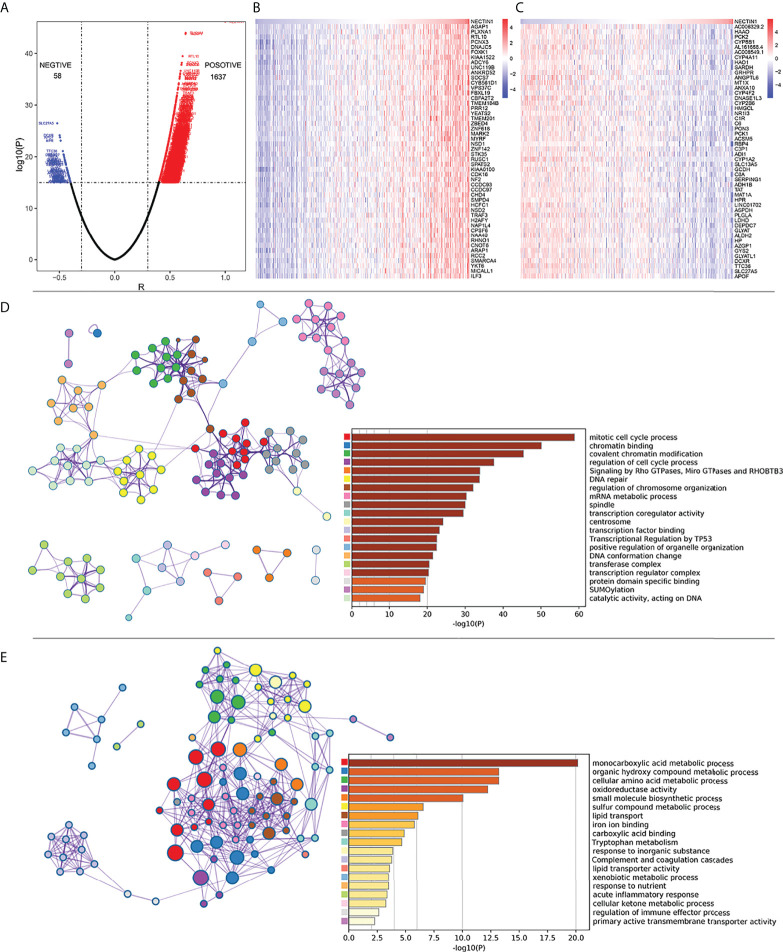
Correlation analysis of coexpressed genes between nectin-1 and HCC tissues. **(A)** Volcano diagram of nectin-1 and coexpressed gene. **(B)** Analysis of the 50 most positively correlated coexpression genes of nectin-1. **(C)** Analysis of 50 most negatively correlated coexpression genes of nectin-1. **(D)** Gene functional enrichment analysis of nectin-1 positively related genes by Metascape. **(E)** Gene functional enrichment analysis of nectin-1-negative correlation gene by Metascape.

Pearson’s correlation analysis was used to analyze the mRNA co-expression of nectin-1 in TCGA-LIHC database. The results showed that there were 1,637 genes positively correlated and 58 genes negatively correlated with nectin-1, respectively (**Figure 5A**). The top 50 positive or top 50 negative coexpression genes of nectin-1 expression are shown in a heatmap (**Figures 5B, C** **)**. Functional enrichment analysis of the positively correlated genes showed that their functions were closely related to mitotic cell cycle processes, chromatin binding, covalent chromatin modification, regulation of cell cycle processes, and signaling by Rho GTPases (**Figure 5D**). Functional enrichment analysis of the negatively correlated genes showed that they were closely related to certain signal pathways, such as the monocarboxylic acid metabolic process, organic hydroxyl compound metabolic process, cellular amino acid metabolic process, and oxidative activity, and to small molecular biological processes, especially the regulation of the immune-effector process (**Figure 5E**).

### Correlation analysis between nectin-1 and immune-related genes in HCC tissue samples

**Figure 6:**
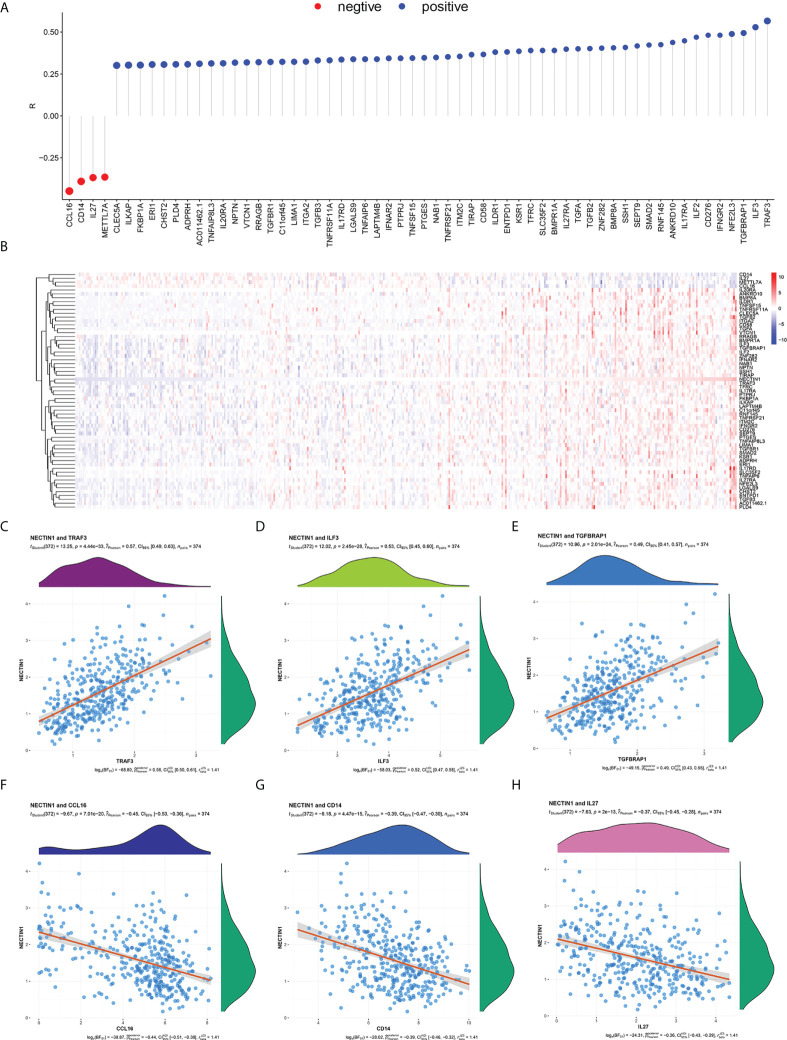
Correlation analysis between nectin-1 and immune coexpressed genes in HCC tissues. **(A)** Analysis of nectin-1 and immune-related coexpression genes. **(B)** Heat map of nectin-1 and immune-related coexpressed genes. **(C)** Correlation between nectin-1 and TRAF-3. **(D)** Correlation between nectin-1 and ILF-3. **(E)** Nectin-1 and TGFBRAP1. **(F)** Correlation between nectin-1 and CCL16. **(G)** Correlation between nectin-1 and CD14. **(H)** Correlation between nectin-1 and IL-27.

Given that the coexpression genes of nectin-1 were related to the regulation of the immune-effector process, we further analyzed the immunity-related genes of nectin-1. The results showed that there were 56 positively related genes and four negatively related genes (**Figure 6A**). The heatmap of the 60 immune-related genes of nectin-1 is shown in **Figure 6B**. Pearson’s correlation analysis showed that the three most positively correlated genes were *TRAF3* (*r* = 0.57, *p* < 0.001), *ILF3* (*r* = 0.53, *p* < 0.001), and *TGFBRAP1* (*r* = 0.49, *p* < 0.001) (**Figures 6C–E**). The three most negatively correlated genes were *CCL16* (*r* = −0.45, *p* < 0.001), *CD14* (*r* = −0.39, *p* < 0.001), and *IL-27* (*r* = −0.37, *p* < 0.001) (**Figures 6F–H**). In addition, nectin-1 was associated with *TGF-β*, *IL-12α*, *IL-10Rα*, and *IL-17Rα*, which are genes that play a key role in immune regulatory function (**Figure 6A**).

### Correlation between nectin-1 to nectin-4 and infiltrating immune cells in HCC tissue samples

**Figure 7:**
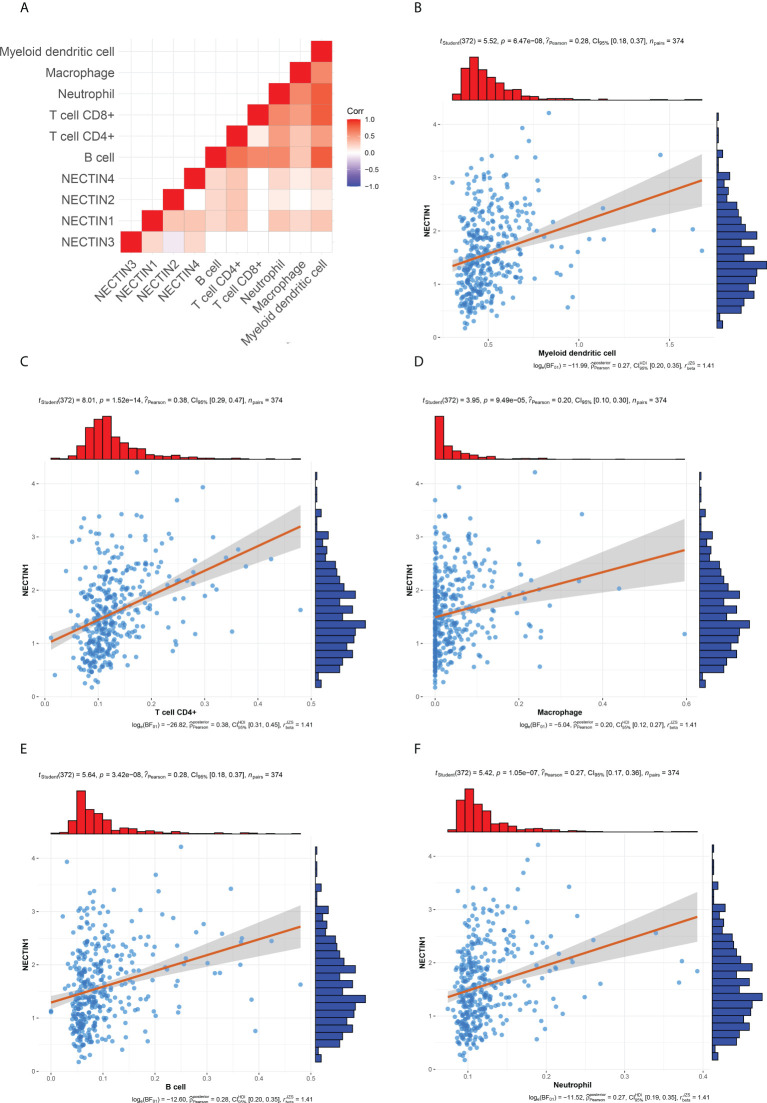
Timer and correlation analysis between nectin-1 and immune cell infiltration in HCC tissues. **(A)** Timer analysis of immune cell infiltration in HCC. **(B)** Correlation analysis between nectin-1 and myeloid DC. **(C)** Correlation between nectin-1 and CD4^+^ T cells. **(D)** Correlation between nectin-1 and macrophage. **(E)** Correlation between nectin-1 and B cells. **(F)** Correlation between nectin-1 and neutrophil cells.

There are many different types of cells in tumor tissues. TIMER can analyze the infiltration by various immune cells in tissue samples (19). The correlation between the nectin family and immune cell infiltration in liver cancer tissues was analyzed by TIMER, and the results showed that nectin-1 positively correlated with B-cell, CD4^+^ T-cell, macrophage, neutrophil, and DC infiltration, while nectin-2 was positively correlated with B-cell, CD4^+^ T-cell, neutrophil, and DC infiltration; nectin-3 did not correlate with any type of immune cell infiltration; and nectin-4 positively correlated with B cell, CD4^+^ T cell, macrophage, neutrophil, and DC infiltration (**Figures 7A, B** **)**. Pearson’s correlation analysis showed that nectin-1 significantly positively correlated with B-cell infiltration (*r* = 0.28, *p* < 0.001), DC infiltration (*r* = 0.28, *p* < 0.001), neutrophil infiltration (*r* = 0.27, *p* < 0.001), CD4^+^ T-cell infiltration (*r* = 0.38, *p* < 0.001), and macrophage infiltration (*r* = 0.20, *p* < 0.001) (**Figures 7C–F**).

### Expression of nectin-1 in HCC cell lines and screening of interfering genes

**Figure 8:**
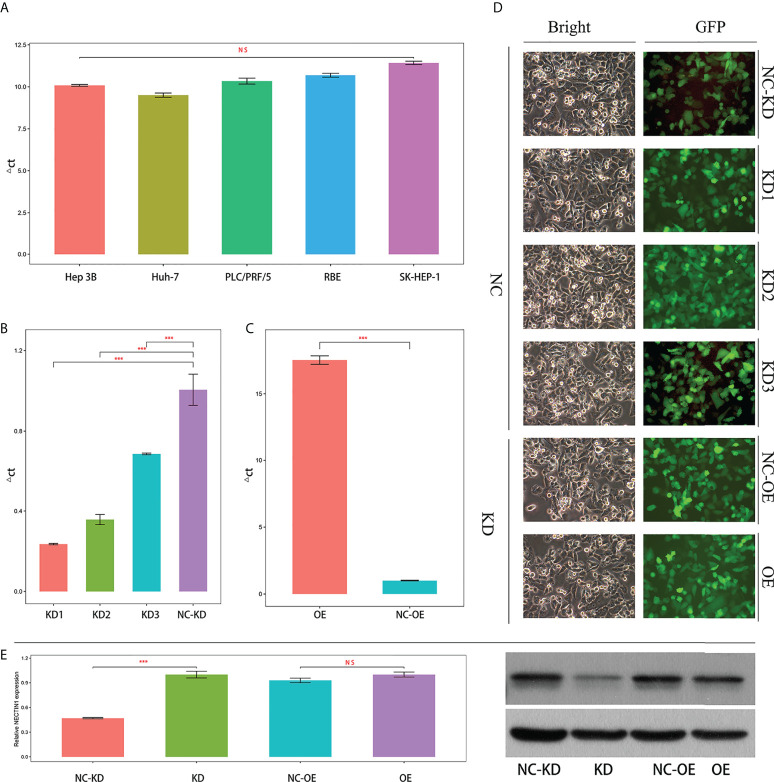
Expression and knockdown screening or overexpression of nectin-1 in the hepatoma cell line. **(A)** Expression of nectin-1 gene in different hepatoma cell lines. **(B)** Knockdown screening of lectin-1 RNA interference in hepatoma cell line. **(C)** Overexpression of nectin-1 gene in hepatoma cell line. **(D)** Expression of GFP in a hepatoma cell line (100×). **(E)** Protein expression after knockdown and overexpression of nectin-1 in the hepatoma cell line. ***P<0.01; ns, no significant.

The expression of nectin-1 in five hepatoma cell lines (SK-Hep-1, Hep 3b, RBE, Huh-7, and PLC/PRF/5) was detected by fluorescence quantitative PCR. The average ΔCT values of nectin-1 (nectin-1-ACTB) in the five hepatoma cell lines were 11.42 ± 0.176, 10.09 ± 0.087, 10.69 ± 0.200, 9.50 ± 0.217, and 10.34 ± 0.302, respectively (the abundance of gene expression was high in the cell when the ΔCT value was ≤12). No significant differences in nectin-1 expression were found among the five hepatoma cell lines (**Figure 8A**). The fluorescence quantitative PCR results of nectin-1 knockout results in SK-Hep-1 cells are shown in **Figure 8B**. The KD efficiency of the nectin-1 gene in the KD1, KD2, and KD3 groups was 76.5%, 64.2%, and 31.5%, respectively; therefore, the KD1 group was selected for nectin-1 gene KD experimentation. According to quantitative PCR analysis, the expression abundance of the nectin-1 gene in the OE group was 17.5 times higher than that in the NC-OE group (**Figure 8C**). The expression of green fluorescence after nectin-1 KD or OE is shown in **Figure 8D**. The protein expression level of nectin-1 decreased significantly after nectin-1 KD in SK-Hep-1 cells; conversely, no significant difference was found after nectin-1 OE in SK-Hep-1 cells (**Figure 8E**).

### Effects of nectin-1 on the proliferation and migration of the hepatoma cell lines

**Figure 9:**
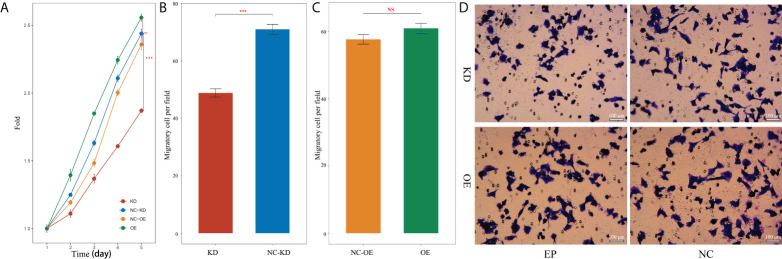
Effects of knockdown and overexpression of nectin-1 on proliferation and migration of hepatoma cells. **(A)** Effects of knockdown and overexpression of nectin-1 on the proliferation of hepatoma cells. **(B)** Effect of knockdown of nectin-1 on the migration of hepatoma cells. **(C)** Effect of overexpression of nectin-1 on the migration of hepatoma cells. **(D)** Cell migration picture of nectin-1 knockdown and overexpression. ***P<0.01; ns, no significant.

The effect of nectin-1 on the proliferation of SK-Hep-1 cells was detected using the CCK-8 kit. The results showed that the cell proliferation of the KD group decreased significantly compared to that of the NC-KD group (*p* < 0.05). The cell proliferation of the nectin-1 in the OE group had no significant change compared to that in the NC-OE group (*p* < 0.05) (**Figure 9A**). A diagram of cell proliferation is shown in **Figure 9B**. A Transwell kit was used to detect the effect of nectin-1 on the migration of SK-Hep-1 cells. The results showed that the number of metastatic cells in the KD group decreased significantly compared to that in the NC-KD group (*p* < 0.05). The number of metastatic cells in the OE group showed no significant differences compared to that in the NC-OE group (*p* > 0.05) (**Figure 9C**). The diagram of cell migration is shown in **Figure 9D**.

## Discussion

As a new group of cell-adhesion molecules, nectins not only mediate cell adhesion but also have a great impact on tumor development and disease progression. The abnormal expression of nectins may play an important role in tumor prognosis (30–32). Here, we showed that the expression levels of the nectin-1 and nectin-2 genes were high and the expression level of the nectin-3 gene was low in HCC tissues compared to that in the adjacent normal tissues. Meanwhile, there was no significant difference in the nectin-4 gene expression. The high expression of the nectin-1 gene correlated with the prognosis of HCC patients according to Kaplan–Meier and Cox regression analyses, which is consistent as a result of that reported by Chiu et al. (33). No significant associations were found between the OS rate and the expression levels of nectin-2, nectin-3, or nectin-4 in HCC patients. However, the expression levels of nectin-1 and nectin-4 proteins from the HPA database were low in HCC tissues, which is inconsistent with the findings of Chiu et al. and Ma et al. (12, 33). This discrepancy might be related to the differences in case selection and sample size. However, we found a significant difference in the expression level of nectin-1 protein between cancer and paracancerous tissues by IHC, consistent with the report by Chiu et al. These findings suggest that nectin-1 may be a potential molecular marker for the prognostic evaluation of HCC patients.

DNA methylation can cause changes in chromatin conformation and DNA stability, thereby affecting gene expression (34). The analysis of the methylation sites of nectins showed that the methylation of nectin-1 negatively correlated with the OS in HCC patients, while the methylation of nectin-4 negatively correlated with the OS of HCC patients. Cox regression analysis showed that nine methylation sites of the nectin-1 gene were closely related to the OS rate of HCC patients, and only one methylation site of the nectin-2 gene was closely related to the OS rate of HCC patients. Two methylation sites of the nectin-3 gene were closely related to the OS rate of HCC patients. Three methylation sites of the nectin-4 gene were closely related to the OS rate of HCC patients. Among the total 72 methylation sites of nectin, 17 sites significantly correlated with the higher OS rate, while 19 methylation sites significantly correlated with the lower OS rate in HCC patients. These results suggest that nectin-1 has many methylation sites, and some of these DNA-methylation sites may influence the prognosis of HCC patients by affecting nectin-1 gene expression.

Immunosuppression and immune escape of tumor cells are important reasons for the development of tumors (3, 35). The interaction mediated by nectin and nectin-like adhesion molecules is crucial for the delicate balance between tumor escape and the antitumor response (36). There are obvious differences in the roles of these molecules in different types of lymphocytes (37). TIMER analysis can reveal the immune cell infiltration of tumor tissues (38). In this study, the results of TIMER analysis showed that nectin-1 positively correlated with B cells, CD4^+^ T cells, macrophages, neutrophils, and DCs, while nectin-2 positively correlated with B cells, CD4^+^ T cells, neutrophils, and DCs, and there was also a positive correlation between nectin-4 and B cells, CD4^+^ T cells, macrophages, neutrophils, and DCs. These results suggest that the abnormal expression of nectin-1, nectin-2, and nectin-4 closely relates to immune cell infiltration in HCC.

Gene functional enrichment analysis of the positively correlated genes of nectin-1 showed that nectin-1 is closely related to the mitotic cell cycle process, chromatin binding, valuable chromatin modification, and regulation of the cell cycle process. Gene functional enrichment analysis of the negatively correlated genes showed that nectin-1 was closely related to the monocarboxylic acid metabolic process, organic hydroxyl compound metabolic process, cellular amino acid metabolic process, oxidative activity, and regulation of the immune-effector process. In particular, nectin-1 was closely related to the regulation of the immune-effector process. Nectin-1 showed a significant association with 60 immune-related genes, including 56 positively related genes and four negatively related genes. Among these related genes, the top three negatively correlated genes were *CCL16*, *CD14*, and *IL-27*, while the top three positively correlated genes were *TRAF3*, *ILF3*, and *TGFBRAP1*. In addition, nectin-1 was associated with *TGF-β*, *IL-12α*, *IL-10RA*, and *IL-17Rα*. Therefore, nectin-1 may interact with multiple immune genes to affect the infiltration and function of immune cells in HCC.

To our knowledge, the effect of nectin-1 on the biological characteristics of HCC cells has not been reported. This study showed that nectin-1 mRNA was highly expressed in hepatoma cell lines. The proliferation of hepatoma cells decreased after KD of nectin-1. However, there was no significant change in the proliferation of hepatoma cells after the OE of nectin-1, which may be related to the high background expression of nectin-1 in hepatoma cells. The number of metastatic cells decreased after the KD of nectin-1 but increased significantly after the OE of nectin-1. These results suggest that nectin-1 may play an important role in the mechanisms of proliferation and metastasis of HCC. The abnormal expression of nectin-1 can effectively distinguish the prognosis of HCC patients with different stages and grades. Checkpoint inhibitors have become an efficient way to treat cancer. The molecules of the “PVR(NECTIN)-TIGIT axis” are a potential target for immune checkpoint therapy (39). These results further prove that inhibitors of nectin-1, along with anti-PD-1 and anti-TIGIT therapies, might be considered for the treatment of HCC. Further experiments *in vivo* should be performed to explore the role of nectin-1 in the metastasis of hepatocellular carcinoma in an animal model. The relationship between the levels of nectin-1 and the prognosis of patients with HCC at different stages should also be analyzed (40, 41).

In conclusion, there is an abnormal expression of nectin-1 and nectin-3 in HCC tissues, and there are multiple methylation sites closely related to the prognosis of HCC patients. The abnormal expression of nectin genes is significantly related to immune cell infiltration and immune-related genes. In particular, nectin-1 can promote the proliferation and migration of liver cancer cells and is closely related to different stages and grades of HCC. Nectin-1 might be a new potential molecular marker for prognostic evaluation as well as a therapeutic target for HCC.

## Data availability statement

The original contributions presented in the study are included in the article/supplementary materials. Further inquiries can be directed to the corresponding authors.

## Ethics statement

The studies involving human participants were reviewed and approved by Taizhou Hospital Affiliated to Wenzhou Medical University. The patients/participants provided their written informed consent to participate in this study.

## Author contributions

TX designed and wrote the manuscript. XW performed the analysis and revised the manuscript. ZX collected data and revised the manuscript. HY conceived of the study and revised the manuscript. HC critically revised the manuscript. QW performed the study. All authors contributed to the article and approved the submitted version.
