# Corrigendum: High expression of nectin-1 indicates a poor prognosis and promotes metastasis in hepatocellular carcinoma

**DOI:** 10.3389/fonc.2023.1134139

**Published:** 2023-03-21

**Authors:** Xuequan Wang, Ziming Xing, Huazhong Chen, Haihua Yang, Qiupeng Wang, Tongjing Xing

**Affiliations:** ^1^ Taizhou Hospital of Zhejiang Province Affiliated to Wenzhou Medical University, Linhai, China; ^2^ Academy of Medical Engineering and Translational Medicine, Tianjin University, Tianjin, China

**Keywords:** hepatocellular carcinoma, nectin-1, prognostic, cell proliferation, metastasis

## Incorrect funding

In the published article, there was an error in the Funding statement [LGH20H190003]. The correct Funding statement appears below.

